# The long-term spatial-temporal trends and burden of esophageal cancer in one high-risk area: A population-registered study in Feicheng, China

**DOI:** 10.1371/journal.pone.0173211

**Published:** 2017-03-07

**Authors:** Xiubin Sun, Deli Zhao, Yi Liu, Yunxia Liu, Zhongshang Yuan, Jialin Wang, Fuzhong Xue

**Affiliations:** 1 Department of Biostatistics, School of Public Health, Shandong University, Jinan, Shandong Province, China; 2 Cancer Research Center, Feicheng People’s Hospital (Tumor Preventative and Therapeutic Base of Shandong Province), Feicheng, Shandong Province, China; 3 Shandong Cancer Hospital, Affiliated to Shandong University, Shandong Academy of Medical Sciences, Jinan, Shandong Province, China; Chinese Academy of Medical Sciences and Peking Union Medical College, CHINA

## Abstract

**Background:**

Feicheng County is a high-risk area for esophageal cancer in Shandong province, China. It is important to determine the long-term spatio-temporal trends in epidemiological characteristics and the burden of esophageal cancer, especially since the implementation of the national esophageal cancer screening program for early detection and treatment in 2005.

**Methods:**

The data collected in Feicheng County from 2001 to 2012 was extracted from the whole-population cancer registry system. The incidence, mortality, disability-adjusted life years (DALY) and changing trends in esophageal cancer according to age and sex were calculated and described.

**Results:**

The incidence rate of esophageal cancer in Feicheng was consistently high, and increased significantly for male, but not for female from 2001 to 2012, according to the joinpoint regression analysis. The highest and lowest yearly crude incidence rates were 160.78 and 95.97 per 100000 for males, and 81.36 and 52.17 per 100000 for females. The highest and lowest crude yearly mortality rates were 122.26 and 94.40 per 100000 for males, and 60.75 and 49.35 per 100000for females. Esophageal squamous cell carcinoma was the main pathology type and the tumor location changed significantly from 2001 to 2012. Overall, the DALY remained roughly stable and was estimated as 11.50 for males and 4.90 for females per 1000 people. The burden was mainly caused by premature death. There is an obvious spatial pattern in the distribution of incidence density and burden.

**Conclusion:**

Esophageal cancer remains a public health issue in Feicheng County with a high incidence, mortality and disease burden. The incidence and burden have obvious spatial heterogeneity, and further studies should be conducted to identify geographical risk factors for precise local prevention and control measures.

## Introduction

Esophageal cancer is a common malignant tumor of the digestive system with a high incidence in many developing countries [1]. China is among the countries with the highest incidence and mortality rate of esophageal cancer, and esophageal squamous cell carcinoma (ESCC) has approximately 90% in rural populations [2]. Esophageal cancer ranks the fifth most common and fourth most lethal malignant tumor in China [3]. The incidence trends of esophageal cancer reported in different studies are inconsistent. Some studies show that the overall incidence rate has significantly decreased [4], while others have shown that it increased slowly from 1989 to 2008 in both urban and rural areas [5, 6,7]. The incidence in rural areas (40.78 and 19.70 cases per 100000 for males and females, respectively) is higher than that in urban regions, as is the mortality rate in China, 2012 [3], and approximately 477900 newly diagnosed esophageal cancer cases were predicted to occur in 2015 in China [8].

Many areas with a prominent high risk of esophageal cancer have been detected in mainland China, including Lin County in Henan Province, Ci County in Hebei Province and Feicheng County in Shandong Province [5,9]. These areas have been the focus of numerous epidemiological studies; however, the incidence trends for esophageal cancer reported in these areas are conflicting [4–7,10]. In addition, even in these small areas, the incidence and mortality of esophageal cancer present obvious spatial-temporal heterogeneity due to geographical environment. For instance, in Ci County, the incidence rate in the mountainous and hilly areas showed a decreasing trend, while in the plains, it remained stable with a slight increase from 1974 to 2002 [11].

In the high-risk area of Feicheng County, esophageal cancer has been a severe public health issue that has led to significant social and financial difficulties [12]. It is the leading cause of cancer death, accounting for 38% of the total deaths caused by all cancers [13]. To reduce the esophageal cancer burden, a national screening program for early detection and treatment has been conducted since 2005. All endoscopic examinations and therapies were conducted by local doctors after receiving training from and while being supervised by experienced doctors from the Cancer Institute. After completion of the informed consent process, the biopsy specimen for each screened participant was obtained using standard protocols, and the biopsy slides were read by two pathologists. When early lesions were histologically diagnosed, the participants were recalled to the clinic, and intervention methods appropriate to the lesions’ severity were used. However, few study have focused on the long-term changes in epidemiological characteristics and the burden of esophageal cancer before and after early screening. In this paper, we present a study investigating the long-term incidence rate, mortality, and burden (combining information for incidence and death) of esophageal cancer in Feicheng County from 2001 to 2012 based on the whole-population registry system. The spatial heterogeneity of esophageal cancer among micro-geographical units is provided.

## Materials and methods

### Data source

Data from the population-based cancer registry in Feicheng County since 1999, together with the corresponding social, geographic, and demographic data, were used to create our spatio-temporal database based on the framework of the geographic information system (GIS) by ArcGIS 9.4. Individual patient information, including demographic characteristics, diagnosis and death data, were obtained following the rules and standards of the International Agency for Research on Cancer (IARC) and the International Association for Cancer Registration. All the data were obtained in a fully anonymized and de-identified manner, and no researchers had access to identifying information. All the cases were diagnosed morphologically and classified by a clinical physician. Classification was based on the ICD10, and some morphological information was incomplete. The WHO Classification of Tumors of the Digestive System was used to classify the tumor stage. Demographic and geographic data were obtained from the Feicheng Municipal Bureau of Statistics. This study was approved by the ethics committee of the School of Public Health, Shandong University.

### Calculation the burden of esophageal cancer

The disability-adjusted life year (DALY) is used to estimate the burden of esophageal cancer. It was designed by the World Health Organization (WHO) to measure, compare and analyze the burden of various diseases [14]. This concept contains two components: years of life lost due to premature mortality (YLLs) and number of years lost due to disability (YLDs). A DALY is equal to the loss of one year of “healthy” life from the combined impacts of mortality and disability.

The YLL corresponds to the number of unlived years in a population because of premature mortality. It is defined as

$$YLL=\frac{KCe^{r\alpha }}{{\left(r+\beta \right)}^{2}}\{e^{-(r+\beta )(L+\alpha )}[-(r+\beta )(L+\alpha )-1]-e^{-(r+\beta )\alpha }[-(r+\beta )\alpha -1]\}+\frac{1-K}{r}{\left(1-e^{-rL}\right)}^{4}$$

where, *K* is an age-weighting modulation factor, *C* is a necessary constant to adjust for unequal age weights, *r* is discount rate, *α* is age at death, *β* is the age-weighting parameter, and *L* is the standard expectation of life at age *α*. The standard life tables at each age and the *YLL* parameters used in the GBD study [15, 16], *K* = 1, *C* = 0.1658, *r* = 0.03, and *β* = 0.04, are also adopted to calculate the *YLLs* of esophageal cancer. The total *YLL*s are further calculated by Σ[(number of deaths due to cause *i* by age and gender)×*YLL*].

*YLD* is defined as

$$YLD=\frac{DKCe^{r\alpha }}{{\left(r+\beta \right)}^{2}}\{e^{-(r+\beta )(L+\alpha )}[-(r+\beta )(L+\alpha )-1]-e^{-(r+\beta )\alpha }[-(r+\beta )\alpha -1]\}+\frac{D(1-K)}{r}{\left(1-e^{-rL}\right)}^{4}$$

where *D* is disability weight, which reflects the severity of each cancer case on a scale from 0 (perfect health) to1 (death), with *D* = 0.20 proposed by the GBD; *α* is the age of onset of the disability; and *L* is duration of disability. *K*, *C*, *r* and *β* are the same as above. Again, the total *YLD*s are further calculated by Σ [(number of incidents due to cause *i* by age and gender)×YLD].

### Statistical analysis

SAS9.0 was used for the data analysis [17]. The changing trend in esophageal cancer was described by the crude rate and age-standardized rate (ASR) of incidence and mortality. The age-standardized rates (ASR) were calculated using data from China's sixth census in 2010. The long-term trends in the ASRs of incidence from 2000 to 2011 were analyzed using the log-transformed joinpoint regression analysis [18]. The annual percent change (APC) in ASRs was also estimated. The APC is based on the assumption that cancer rates change at a constant percentage of the previous year’s rate, and it was tested to determine if it differed from the null hypothesis that the APC was equal to zero. DisMod (disease-modeling) software [14] was used to calculate the *YLL*s, *YLD*s and DALY; it provides an internally consistent set of epidemiological indices, including incidence, prevalence, remission, duration, mortality, case fatality, and relative risk of mortality for diseases and injuries. Categorical variables were compared using the chi-square test or Fisher’s exact test. *P* values less than 0.05 were considered statistically significant.

## Result

### Epidemiological characteristics of esophageal cancer from 2001 to 2012

There were 8431 cases of incident esophageal cancer diagnosed from 2001 to 2012 in Feicheng County. Of these, 5638 patients were males (66.88%), and 2793 were females (33.12%). The age at final diagnosis for the males was 62±10 years old (the youngest age final diagnosis was 18 and the oldest was 92 years); for the females, the age at final diagnosis was 66±11 years (the youngest was 20 and the oldest was 97 years old). Of the cases, 1995 had an uncertain tumor classification, and 2550 had no clear tumor locations.

#### Incidence rate

**Fig 1:**
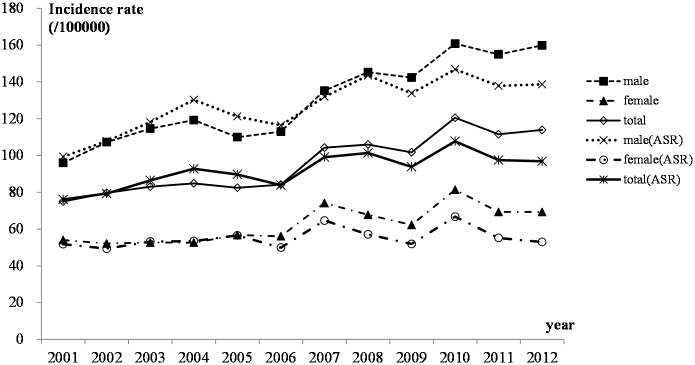
Incidence of esophageal cancer from 2001–2012 in Feicheng County.

The overall crude rate and age-adjusted rate of esophageal cancer incidence in Feicheng were quite high. The rates dropped between 2001 and 2005 and then increased each year since 2006, though the growth was slow (Fig 1), and the changing trend was significantly for males, while not for females, according to the joinpoint regression analysis. The joinpoint analysis also showed that there was only one segment from 2001 to 2012 for both males and females: the APC for males increased by 2.77 (P<0.05) per year between 2001 and 2012, while for females, the APC was 1.12, which was not significant (P = 0.2). Moreover, the incidence rate for the males was nearly twice as high as for the females. The highest and lowest crude incidence rates for the males were 160.78 and 95.97 per 100000, respectively, while for females, they were 81.36 and 52.17 per 100000, respectively. After the standardization of the population’s age structure with the data from China's sixth census in 2010, the highest and lowest age-standardized incidence rates for different years for males were 146.86 and 99.35 per 100000, respectively, while for the females, the rates were 66.75 and 49.34 per 100000, respectively.

The median survival times for males and females were 14.93 months and 15.60 months, respectively; the 95% confidence intervals were 14.43–15.43 months and 14.67–16.37 months for males and females, respectively.

**Fig 2:**
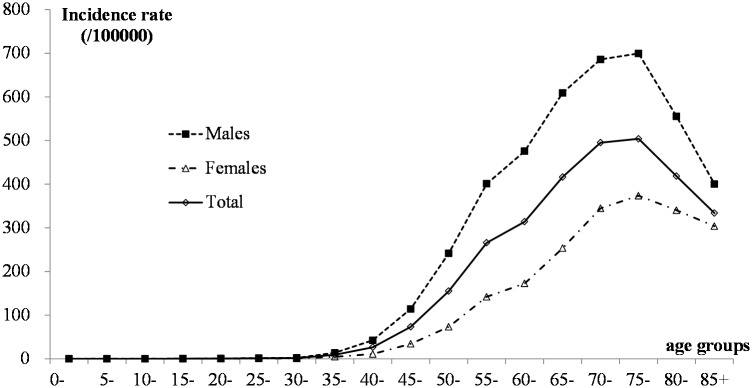
Age-specific changes in the incidence of esophageal cancer in Feicheng County from 2001–2012.

To study the incidence at different ages, we calculated the incidence rates by age group, and Fig 2 showed the changes in age-specific incidence rates. For both males and females, the incidence rate increased significantly after the age of 45 years, and the peaks were among those over 60 years old, especially those over 70 years old.

#### Mortality rate

**Fig 3:**
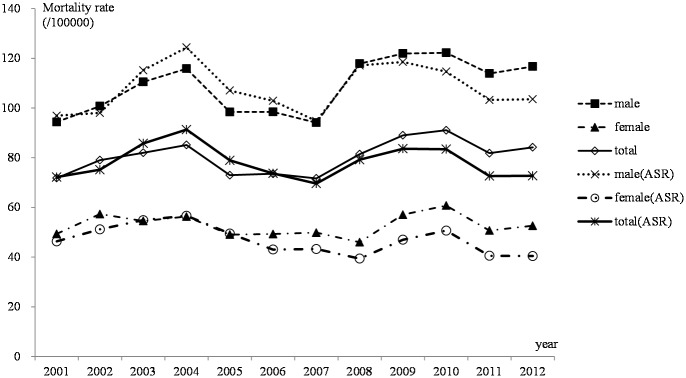
Mortality of esophageal cancer from 2001–2012 in Feicheng County.

Considering that esophageal cancer was a rapidly developing and highly lethal disease, mortality rates were comparable to the incidence rates. The mortality trend for esophageal cancer in rural areas of Feicheng County was relatively stable from 2001–2012, and there was almost no change in mortality among females. The highest and lowest yearly crude mortality rates for males were 122.26 and 94.40 per 100000, respectively, from year 2001 to 2012; for females, the rates were 60.75and 49.35 per 100000, respectively. After standardization of the population’s age structure using data from China's sixth census in 2010, the highest and lowest age-standardized incidence rates per calendar year for males were 124.39 and 94.77 per 100000, respectively, while those for females were 56.64 and 39.41 per 100000, respectively (Fig 3).

#### Tumor types, locations and differentiation

Among all the patients, 6436 (4424 males and 2012 females) had pathological tumors; the classification of the others was uncertain. Of the patients diagnosed with a definite pathological type, esophageal squamous cell carcinoma (ESCC) accounted for 6376 cases or approximately 99.07% of the total and was the main pathologic type; adenocarcinoma accounted for 60 cases or approximately 0.93%. For both males and females, the proportion of the two pathological types had almost no difference in different years (*P* values from Fisher’s exact test were 0.150 and 0.189 for males and females, respectively). Hence, there was no difference between the males and females (*χ*^2^ = 0.2417 and *P* = 0.6230).

The case-fatality was associated with the stage at which the tumor was diagnosed. The stage at which the tumor was diagnosed was reported for 4436 patients. Only approximately 6.5% of the tumors were detected in the stage I, while 17.3%, 75.4% and 0.8% of tumors were diagnosed in the stage II, stage III and stage IV, respectively. These result show that the tumors were primarily detected at the middle and late stages of cancer, and there was no difference between the males and females (*χ*^2^ = 2.5280 and *P* = 0.4702). The proportion of esophageal cancer cases detected in the 3rd stage rose from 2001–2012, but the proportions of tumors detected in the 1st and 2nd stages fell from 2001 to 2012. The trends did not differ between the males and females.

**Fig 4:**
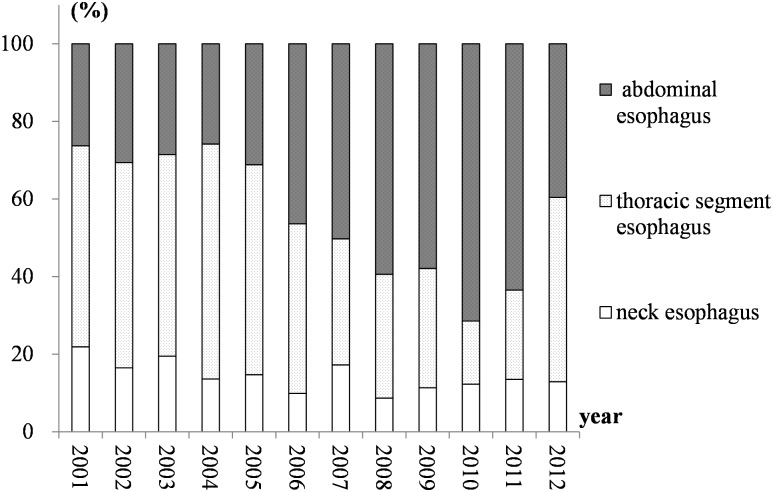
The proportions for the different locations between 2001 and 2012 in Feicheng County.

Definite tumor locations were reported for 5881 patients (3945 males and 1936 females). Tumors in the neck esophagus, the thoracic segment esophagus and the abdominal esophagus accounted for approximately 14.8%, 41.7% and 43.5% of all reported cases, respectively. We compared the proportions at different sites and found that the proportions of tumors at different locations did not differ between the males and females (*χ*^2^ = 2.1882 and *P* = 0.3348). Fig 4 showed the change in proportions after the male and female subgroups were merged. The proportion of esophageal cancer in the abdominal esophagus increased from 2001 to 2010 and then declined from 2011 to 2012. In contrast, for tumors in the thoracic segment esophagus, the proportion decreased from 2001 to 2010 but increased in the following two years. The proportion of tumors in the neck esophagus showed no obvious change from 2001 to 2012, and the trends for males and females did not differ.

### Disease burden of esophageal cancer from 2001 to 2012

#### Total burden of esophageal cancer in Feicheng County

The burden of esophageal cancer by sex revealed differences between males and females in different years in Feicheng County (Table 1). The results showed that the burden had a small change from 2001 to 2012. Its peak was in 2010, but the overall disease burden remained roughly stable each year. Males had higher DALYs and DALY rates compared with females; the total burden was estimated to be 49924.00 DALYs for males and 22032.00 for females from 2001 to 2012, with DALY rates per 1000 people of 11.50 for males and 4.90 for females.

**Table 1:** Burden for esophageal cancer in different years in Feicheng County from 2006–2012.

Year	YLLs		YLDs		DALYs		DALYs per 1000		
	Males	Females	Males	Females	Males	Females	Males	Females	Total
2001	3520.94	1660.07	251.03	133.94	3771.98	1794.01	9.89	4.68	7.28
2002	3715.08	1742.27	268.30	138.29	3983.38	1880.56	10.32	4.86	7.58
2003	3700.07	1762.03	270.93	136.73	3970.99	1898.76	10.91	5.03	7.92
2004	3697.16	1752.24	278.13	134.39	3975.29	1886.63	11.20	4.99	8.00
2005	3474.37	1543.92	283.42	141.13	3757.79	1685.05	10.57	4.45	7.41
2006	3382.49	1547.50	285.87	139.47	3668.36	1686.97	10.26	4.59	7.39
2007	3317.89	1513.78	327.72	166.38	3645.61	1680.16	10.21	4.58	7.36
2008	4230.35	1378.06	357.13	174.98	4587.47	1553.04	12.84	4.23	8.47
2009	4199.94	1807.49	376.54	186.07	4576.48	1993.55	12.80	5.42	9.06
2010	4315.88	1825.19	409.53	210.82	4725.41	2036.01	13.19	5.52	9.30
2011	3995.06	1421.08	429.97	211.97	4425.03	1633.06	12.29	4.40	8.29
2012	3928.17	1575.87	462.79	227.47	4390.96	1803.34	12.15	4.84	8.44
Total	45477.00	19529.51	4447.00	2503.00	49924.00	22032.00	11.50	4.90	8.10

Based on the total number of DALYs, the burden of esophageal cancer due to premature mortality (YLLs) was 45477.00, accounting for 91.09% of the total burden for males; for females, the burden was 19529.51, accounting for 88.64% of the total burden (Table 1). Our study indicated that the burden of esophageal cancer was primarily caused by premature death.

The changes in DALYs and DALY rates for females were mild; in general, the trends were relatively stable from 2001 to 2012. In contrast, the burden for males, which was higher than that for females, increased considerably after 2007. Moreover, the DALY rates rose from near 10 to as high as 13. The highest and lowest DALY rates were 13.19 and 9.89, respectively, for males and 5.52 and 4.23, respectively, for females.

#### Burden of esophageal cancer for different age groups

Table 2 showed the burden of esophageal cancer for different age groups. The burden was concentrated at the ages of 40–80 years, and the general trend for DALYs increased with age. Patients aged between 40 and 80 years accounted for 95.20% of the total DALYs, and the proportions for males and females were 96.21% and 92.92%, respectively.

**Table 2:** The burden of esophageal cancer in different age groups in Feicheng County from 2001–2012.

Age	DALYs			DALYs per 1000		
	Males	Females	Total	Males	Females	Total
0-	0.00	0.00	0.00	0.00	0.00	0.00
15-	36.00	0.00	36.00	0.10	0.00	0.10
20-	34.00	34.00	68.00	0.10	0.10	0.10
25-	93.00	31.00	125.00	0.30	0.10	0.20
30-	98.00	57.00	155.00	0.30	0.20	0.20
35-	598.00	177.00	775.00	1.70	0.50	1.10
40-	1985.00	746.00	2731.00	5.00	1.90	3.40
45-	5666.00	1324.00	6990.00	15.60	3.50	9.40
50-	8432.00	2765.00	11196.00	26.70	8.30	17.20
55-	10970.00	3908.00	14878.00	41.00	13.40	26.60
60-	7760.00	3496.00	11257.00	40.40	16.00	27.40
65-	6409.00	3122.00	9531.00	48.40	20.00	33.00
70-	4344.00	3095.00	7439.00	44.10	24.80	33.30
75-	2464.00	2016.00	4480.00	36.00	19.70	26.20
80-	847.00	943.00	1790.00	22.60	14.40	17.40
85-	187.00	318.00	506.00	10.10	7.70	8.50
Total	49924.00	22032.00	71956.00	11.50	4.90	8.10

The DALYs rate was 8.10 for the total population and 11.50 and 4.90 for males and females, respectively. The peak DALYs rate was focused primarily on people aged 55 to 80 years. The highest DALYs rate for males was 48.40 among those aged 65 to 70 years; for females, it was 24.80 for those between 70 and 75 years.

### Spatial distribution of esophageal cancer in Feicheng County

**Fig 5:**
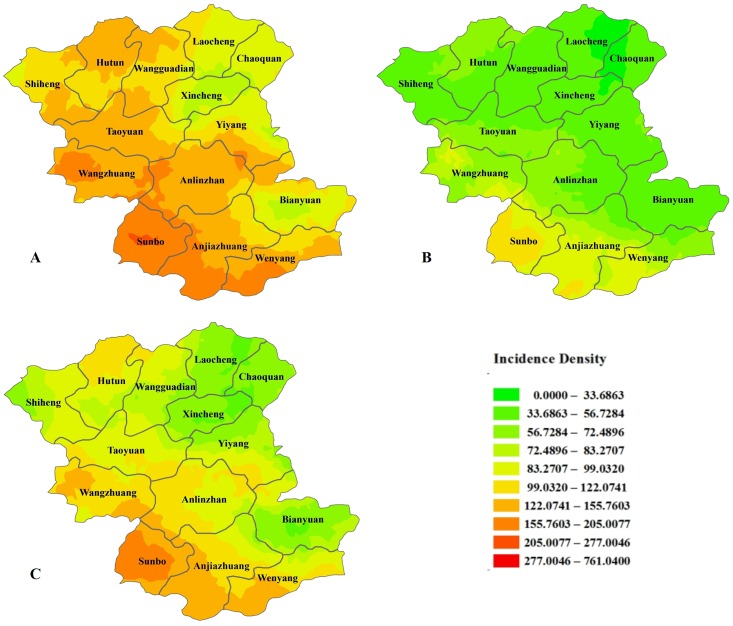
The spatial distribution of the incidence density of disease in Feicheng County. Figures A, B, and C are the spatial distributions of the male, female and total incidence densities, respectively.

**Fig 6:**
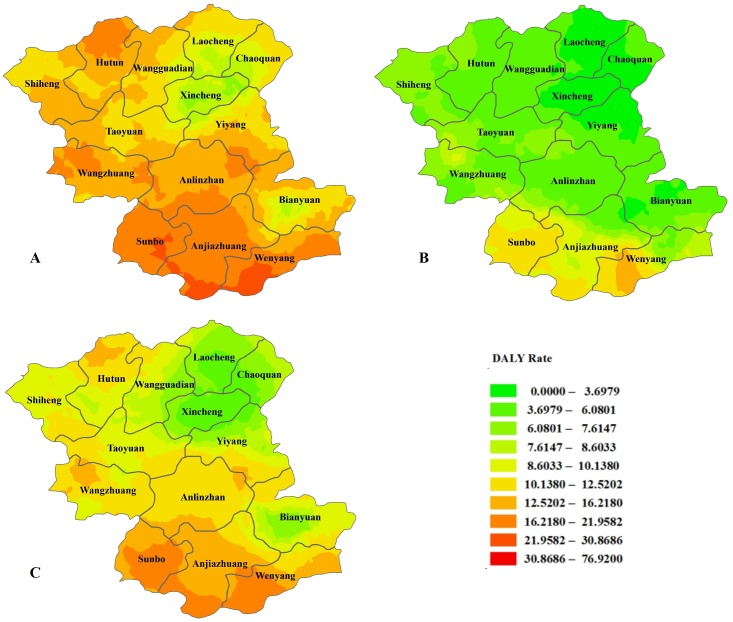
The spatial distribution of the burden of disease in Feicheng County. Figures A, B, and C are the spatial distributions of the male, female and total DALY rates, respectively.

Feicheng is located in central Shandong Province on the west wing of Mount Taishan. The geographic coordinates for latitude are from 35 degrees 53 minutes to 36 degrees 19 minutes, and the eastern longitude is from 116 degrees 28 minutes to 116 degrees 59 minutes. The total area is 1277.3 square kilometers, and the county comprises 609 administrative villages. We calculated the incidence density and disease burden of esophageal cancer for the village as a unit and drew a sex-specific statistical map. Figs 5 and 6 show the spatial distribution of the incidence density and burden of disease, which indicates an obvious tendency toward high density in the lower-altitude southwestern area and a lower density in the higher-altitude northeastern area.

## Discussion

In the present study, we investigated the long-term epidemiological characteristics and the burden of esophageal cancer based on the whole-population registry system for esophageal cancer in Feicheng County. In China, the incidence rates of esophageal cancer for males and females in rural populations are 40.78 and 19.70 per 100000, respectively, and the mortality rates are 29.30 and 14.44 per 100000, respectively [3]. As expected, the incidence rate and mortality in Feicheng County are obviously higher than the national average level for both males and females, and they are nearly twice as high for males as for females. The incidence rate showed a slightly increasing trend from 2001 to 2004 and then increased sharply each year from 2005 to 2012; however, the change in the trend was not significant according to the joinpoint regression analysis. Consistent with previous studies [2], squamous cell carcinoma was the main type of esophageal cancer in Feicheng County. The predominant tumor location appears to have changed from 2001 to 2012. The proportion of tumors in the abdominal esophagus increased from 2001 to 2010 and then decreased starting in 2011. Although early esophageal cancer screening has been conducted since 2005, most esophageal cancer cases are still identified in the advanced stage, suggesting that the screening program is not adequate for detecting esophageal cancer at an early stage. The trends in the changes in tumor location and clinical stage after the implementation of screening have not been previously reported. Studies on the reasons for these changes could have a positive impact on reducing the incidence of esophageal cancer in this high-risk area.

In the study region, the DALYs rate (8.10 per 1000 people) is far higher than the average burden (2.06 per 1000 people) for rural residents in Shandong Province [19]. Similar to previously reported results, our finding shows that YLL is the predominant contributor to DALY estimates because esophageal cancer is a fatal disease. In fact, the burden of fatal diseases mainly results from premature death, even for cancer, a disease for which the number of incident cases is approximately twice the number of deaths. A study in France reported that YLLs contributed to 98% of the DALYs for lung cancer in men and 86% of the DALYs for breast cancer in women [20], and a study in Australia showed that YLLs contributed more 80% of the total DALYs for all cancers [21]. Another study estimating the cancer burden in Spain in 2000 reported that YLLs contributed to 84% of the DALYs for cancer [22]. In contrast, for less fatal diseases, the impact of YLDs might be greater than that of YLLs; for instance, the DALYs for mental disorders and musculoskeletal disease in Australia was primarily attributable to YLDs [23].

The topography of Feicheng County is complex and diverse. It contains plains, mountains and hills; the terrain is tilted from northeast to southwest; the highest point is 600 meters; and the lowest point is 57.7 meters above sea level. Environmental exposure is associated with esophageal cancer, and even in this small area, the changes in the incidence rate of esophageal cancer were different because of different geographical environments [11]. Some previous studies have found a positive relationship between aflatoxin levels in wheat flour and selenium levels in soil and rice and the risk of esophageal cancer [24, 25]. Other studies have shown that the incidence of esophageal cancer is associated with elevation, suggesting that high altitude might reduce the incidence of esophageal cancer [26]. Our study showed that the incidence rate and the burden of disease are higher in the southwest than that in the northeast region of Feicheng County. This result provides a guideline for allocating prevention and control measures based on the geography of this area.

Various case–control and cohort studies have been performed to detect risk factors for esophageal cancer. It has been indicated that the development of esophageal cancer is frequently induced by chronic exposure to irritants, spices, hot drinks, alcohol, and smoking. The risk factors for esophageal squamous cell carcinoma differ from those of esophageal adenocarcinoma [27]. The risk factors for the adenocarcinomas mainly include obesity, gastroesophageal reflux disease and some dietary factors, such as diets high in total fat, saturated fat and cholesterol [28,29], while tobacco smoking and alcohol consumption are the most important risk factors for squamous cell carcinoma [29,30].

In conclusion, esophageal cancer is still a severe public health issue in Feicheng County with a high incidence, mortality and disease burden, despite the national screening program for early detection and treatment that has been in place since 2005. In addition, both incidence and mortality present high spatial heterogeneity even at the village level in this small area, which indicates that micro-geographical risk factors for esophageal cancer might exist in high-incidence villages. Therefore, it is important to conduct further studies to identify the geographical risk factors and determine precise local prevention and control measures.

## Supporting information

S1 Appendix Dataset for all figures. (XLSX)
